# Supplementary material for: Navigable maps of structural brain networks across species
Source: PLoS Comput Biol. 2020 Feb 3;16(2):e1007584. doi: 10.1371/journal.pcbi.1007584 (PMC7018228; doi:10.1371/journal.pcbi.1007584)
Supplement: S1 Table — Also shown are estimations of the 95% confidence interval obtained by 10000 resampling of the original data. (PDF) [file pcbi.1007584.s001.pdf]

Dataset	Pearson correlation coefficient	Spearman's rank correlation coefficient
Human1	0.910 (0.906, 0.914)	0.847 (0.841, 0.852)
Human2	0.864 (0.857, 0.871)	0.826 (0.820, 0.833)
Human3	0.494 (0.483, 0.504)	0.501 (0.489, 0.513)
Human4	0.507 (0.499, 0.516)	0.542 (0.532, 0.551)
Human5	0.478 (0.473, 0.483)	0.527 (0.521, 0.533)
