# Supplementary material for: Navigable maps of structural brain networks across species
Source: PLoS Comput Biol. 2020 Feb 3;16(2):e1007584. doi: 10.1371/journal.pcbi.1007584 (PMC7018228; doi:10.1371/journal.pcbi.1007584)
Supplement: S2 Table — (PDF) [file pcbi.1007584.s002.pdf]

**Dataset**	**Pearson correlation coefficient**
Drosophila1	0.215
Drosophila2	0.145
ZebraFinch1	0.056
CElegans	0.384
Mouse1	0.167
Mouse2	0.460
Macaque1	0.501
Macaque4	0.371
Human1	0.042
Human2	0.049
Human3	0.170
Human4	0.288
Human5	0.192
Human8	0.561
