# Supplementary material for: Navigable maps of structural brain networks across species
Source: PLoS Comput Biol. 2020 Feb 3;16(2):e1007584. doi: 10.1371/journal.pcbi.1007584 (PMC7018228; doi:10.1371/journal.pcbi.1007584)
Supplement: S1 Appendix — (PDF) [file pcbi.1007584.s003.pdf]

OVERVIEW OF THE DIFFERENT DATASETS

Name	Node	Zone	Pos.	N	L	ρ	$\langle k \rangle$	r	$\bar{c}$	Ref.
C. Elegans	cell	nervous system	yes	279	2287	0.060	16.4	-0.09	0.34	[1, 2][1]
Cat1	area	cortex	no	65	730	0.351	22.5	-0.03	0.66	[3, 4][2]
Cat2	area	cortex and thalamus	no	95	1170	0.262	24.6	-0.09	0.62	[5][3]
Cat3	area	cortex	no	52	515	0.388	19.8	-0.04	0.66	[5][3]
Drosophila1	cell	optic medulla	yes	350	2887	0.047	16.5	-0.02	0.24	[6][4]
Drosophila2	cell	optic medulla	yes	1781	8911	0.006	10.0	-0.09	0.26	[6][5]
Macaque1	area	cortex	yes	94	1515	0.347	32.2	-0.15	0.77	[7][6]
Macaque2	area	cortex	no	71	438	0.176	12.3	0.09	0.50	[8][7]
Macaque3	area	cortex	no	242	3054	0.105	25.2	-0.05	0.45	[9][8]
Macaque4	area	cortex	yes	29	322	0.793	22.2	-0.15	0.87	[10, 11][9]
Mouse1	area	whole brain	yes	213	2969	0.132	27.9	-0.05	0.45	[12][10]
Mouse2	cell	retina	yes	916	77585	0.185	169.4	-0.18	0.60	[13][11]
Mouse3	cell	retina	no	1076	90811	0.157	168.8	-0.20	0.59	[13][11]
Human1	area	cortex (1 hemishpere)	yes	493	7773	0.064	31.5	0.21	0.49	[14, 15][12]
Human2	area	cortex (1 hemisphere)	yes	496	8037	0.065	32.4	0.25	0.48	[14, 15][12]
Human3	area	cortex (2 hemispheres)	yes	256	9103	0.279	71.1	0.09	0.68	[16, 17][13]
Human4	area	cortex (2 hemispheres)	yes	360	12100	0.187	67.2	0.05	0.66	[16, 17][13]
Human5	area	cortex (2 hemispheres)	yes	1024	36553	0.070	71.4	0.09	0.60	[16, 17][13]
Human6	area	whole brain	no	110	966	0.161	17.6	0.20	0.61	[18][14]
Human7	area	whole brain	no	116	1165	0.175	20.1	0.27	0.63	[18][15]
Human8	area	whole brain	yes	246	11060	0.367	89.9	-0.01	0.69	[19][16]
Rat1	area	nervous system	no	503	23030	0.182	91.6	-0.73	0.89	[20][17]
Rat2	area	nervous system	no	502	24647	0.196	98.2	-0.74	0.89	[20][18]
Rat2	area	nervous system	no	493	25979	0.214	105.4	-0.70	0.88	[20][19]
ZebraFinch1	cell	basal-ganglia (Area X)	yes	582	2210	0.013	7.6	-0.16	0.04	[21][20]
ZebraFinch2	cell	basal-ganglia (Area X)	no	610	15342	0.083	50.3	-0.26	0.11	[21][21]

TABLE I. Overview of the different datasets. For each connectome, the definition of nodes, the zone of the brain covered as well as whether geometrical information (i.e., positions) is available is indicated. The number of nodes (N), the number of links (L), the density of links ($\rho = 2L/N(N-1)$), its average degree ($\langle k \rangle = 2L/N$), the assortativity coefficient (r) and the average local clustering coefficient ($\bar{c}$).

Table footnotes

1. The 3D positions were downloaded from Yong-Yeol Ahn's professional website (http://yongyeol.com/data/celegans.net) and the connectivity data were downloaded from http://www.wormatlas.org/images/NeuronConnect.xls.

2. Downloaded from the NeuroData's Graph DataBase (https://neurodata.io/project/connectomes): https://s3.amazonaws.com/connectome-graphs/cat/mixed.species_brain_1.graphml.

3. Dataset is hosted on the Brain Connectivity Toolbox website (https://sites.google.com/site/bctnet/datasets): https://sites.google.com/site/bctnet/Home/functions/cat.mat.

4. Extracted from Supplementary Table 1 of Ref. [6].

5. Downloaded from the NeuroData's Graph DataBase (https://neurodata.io/project/connectomes): https://s3.amazonaws.com/connectome-graphs/fly/drosophila_medulla_1.graphml.

6. Downloaded from Dynamic Connectome Lab website (https://www.dynamic-connectome.org/): https://www.dynamic-connectome.org/pubs/suppl/mac95.mat.

7. Downloaded from the Brain Connectivity Toolbox website (https://sites.google.com/site/bctnet/datasets): https://sites.google.com/site/bctnet/Home/functions/macaque71.mat.

8. Downloaded from the NeuroData's Graph DataBase (https://neurodata.io/project/connectomes): https://s3.amazonaws.com/connectome-graphs/macaque/rhesus_brain_1.graphml.

9. Based on information provided by the authors of Ref. [16], the connectivity data were extracted from the supplementary data of Ref. [11] (can be downloaded in xls formal from http://core-nets.org/download/Cercor_2012%20Table.xls) and the distances published alongside Ref. [10] (can be downloaded in xls format from http://core-nets.org/download/PNAS_2013_Distance_Matrix.xlsx).

10. The distances were extracted from Supplementary Table 4 from Ref. [12] and the connectivity data were kindly provided by the authors of Ref. [12].

11. Downloaded from the NeuroData's Graph DataBase (https://neurodata.io/project/connectomes): https://s3.amazonaws.com/connectome-graphs/mouse/mouse_retina_1.graphml. Mouse2 consists in the subset of Mouse3 for which spatial coordinates of the nodes were provided.

12. Downloaded from https://ndownloader.figshare.com/files/10446582. Human1 and Human2 correspond to the left and right hemispheres, respectively.

13. Dataset was kindly provided by the authors of Ref. [16].

14. Downloaded from http://openconnecto.me/mrdata/share/dti/ndmg_v0011/NKIENH/AAL/NKIENH_0197456_1_DTI_AAL.graphml.

15. Downloaded from http://openconnecto.me/mrdata/share/dti/ndmg_v0011/NKIENH/HarvardOxford/NKIENH_0198051_1_DTI_HarvardOxford.graphml.

16. Extracted from files `BNA_matrix_binary_246x246.csv`, `BNA_subregions.xlsx` and `subregion_func_network_Yeo.csv` downloaded from http://atlas.brainnetome.org/download.html.

17. Downloaded from the NeuroData's Graph DataBase (https://neurodata.io/project/connectomes): https://s3.amazonaws.com/connectome-graphs/rat/rattus.norvegicus_brain_1.graphml.

18. Downloaded from the NeuroData's Graph DataBase (https://neurodata.io/project/connectomes): https://s3.amazonaws.com/connectome-graphs/rat/rattus.norvegicus_brain_2.graphml.

19. Downloaded from the NeuroData's Graph DataBase (https://neurodata.io/project/connectomes): https://s3.amazonaws.com/connectome-graphs/rat/rattus.norvegicus_brain_3.graphml.

20. Dataset was kindly provided by the authors of Ref. [21].

21. Connectivity data were extracted from Supplementary Figure 7 of Ref. [21] (an excel file is provided on the corresponding Nature Methods page).

[1] Y.-Y. Ahn, H. Jeong, and B. J. Kim, "Wiring cost in the organization of a biological neuronal network," Physica A **367**, 531–537 (2006).

[2] L. R. Varshney, B. L. Chen, E. Paniagua, D. H. Hall, and D. B. Chklovskii, "Structural Properties of the Caenorhabditis elegans Neuronal Network," PLoS Comput. Biol. **7**, e1001066 (2011).

[3] J. W. Scannell, C. Blakemore, and M. P. Young, "Analysis of connectivity in the cat cerebral cortex," J. Neurosci. **15**, 1463–83 (1995).

[4] M. A. de Reus and M. P. van den Heuvel, "Rich Club Organization and Intermodule Communication in the Cat Connectome," J. Neurosci. **33**, 12929–12939 (2013).

[5] J. W. Scannell, "The Connectional Organization of the Cortico-thalamic System of the Cat," Cereb. Cortex **9**, 277–299 (1999).

[6] S.-Y. Takemura, A. Bharioke, Z. Lu, A. Nern, S. Vitaladevuni, P. K. Rivlin, W. T. Katz, D. J. Olbris, S. M. Plaza, P. Winston, T. Zhao, J. A. Horne, R. D. Fetter, S. Takemura, K. Blazek, L.-A. Chang, O. Ogundeyi, M. A. Saunders, V. Shapiro, C. Sigmund, G. M. Rubin, L. K. Scheffer, I. A. Meinertzhagen, and D. B. Chklovskii, "A visual motion detection circuit suggested by Drosophila connectomics," Nature **500**, 175–181 (2013).

[7] M. Kaiser and C. C. Hilgetag, "Nonoptimal Component Placement, but Short Processing Paths, due to Long-Distance Projections in Neural Systems," PLoS Comput. Biol. **2**, e95 (2006).

[8] M. P. Young, "The Organization of Neural Systems in the Primate Cerebral Cortex," Proc. R. Soc. B **252**, 13–18 (1993).

[9] L. Harriger, M. P. van den Heuvel, and O. Sporns, "Rich Club Organization of Macaque Cerebral Cortex and Its Role in Network Communication," PLoS One **7**, e46497 (2012).

[10] N. T. Markov, M. Ercsey-Ravasz, C. Lamy, A. R. Ribeiro Gomes, L. Magrou, P. Misery, P. Giroud, P. Barone, C. Dehay, Z. Toroczkai, K. Knoblauch, D. C. Van Essen, and H. Kennedy, "The role of long-range connections on the specificity of the macaque interareal cortical network," Proc. Natl. Acad. Sci. USA **110**, 5187–5192 (2013).

[11] N. T. Markov, M. M. Ercsey-Ravasz, A. R. Ribeiro Gomes, C. Lamy, L. Magrou, J. Vezoli, P. Misery, A. Falchier, R. Quilodran, M. A. Gariel, J. Sallet, R. Gamanut, C. Huissoud, S. Clavagnier, P. Giroud, D. Sappey-Marinier, P. Barone, C. Dehay, Z. Toroczkai, K. Knoblauch, D. C. Van Essen, and H. Kennedy, "A Weighted and Directed Interareal Connectivity Matrix for Macaque Cerebral Cortex," Cereb. Cortex **24**, 17–36 (2014).

[12] S. W. Oh, J. A. Harris, L. Ng, B. Winslow, N. Cain, S. Mihalas, Q. Wang, C. Lau, L. Kuan, A.M. Henry, M. T. Mortrud, B. Ouellette, T. N. Nguyen, S. A. Sorensen, C. R. Slaughterbeck, W. Wakeman, Y. Li, D. Feng, A. Ho, E. Nicholas, K. E. Hirokawa, P. Bohn, K. M. Joines, H. Peng, M. J. Hawrylycz, J. W. Phillips, J. G. Hohmann, P. Wohnoutka, C. R. Gerfen, C. Koch, A. Bernard, C. Dang, A. R. Jones, and H. Zeng, "A mesoscale connectome of the mouse brain," Nature **508**, 207–214 (2014).

[13] M. Helmstaedter, Kevin L. Briggman, Srinivas C. Turaga, V. Jain, H. S. Seung, and Winfried Denk, "Connectomic reconstruction of the inner plexiform layer in the mouse retina," Nature **500**, 168–174 (2013).

[14] A. Avena-Koenigsberger, J. Goni, R. F. Betzel, M. P. van den Heuvel, A. Griffa, P. Hagmann, J.-P. Thiran, and O. Sporns, "Using Pareto optimality to explore the topology and dynamics of the human connectome," Philos. Trans. R. Soc. B Biol. Sci. **369**, 20130530 (2014).

[15] P. Hagmann, L. Cammoun, X. Gigandet, R. Meuli, C. J. Honey, V. J. Wedeen, and O. Sporns, "Mapping the Structural Core of Human Cerebral Cortex," PLoS Biol. **6**, e159 (2008).

[16] C. Seguin, M. P. van den Heuvel, and A. Zalesky, "Navigation of brain networks." Proc. Natl. Acad. Sci. U. S. A. **115**, 6297–6302 (2018).

[17] D.C. Van Essen, K. Ugurbil, E. Auerbach, D. Barch, T.E.J. Behrens, R. Bucholz, A. Chang, L. Chen, M. Corbetta, S.W. Curtiss, S. Della Penna, D. Feinberg, M.F. Glasser, N. Harel, A.C. Heath, L. Larson-Prior, D. Marcus, G. Michalareas, S. Moeller, R. Oostenveld, S.E. Petersen, F. Prior, B.L. Schlaggar, S.M. Smith, A.Z. Snyder, J. Xu, and E. Yacoub, "The Human Connectome Project: A data acquisition perspective," NeuroImage **62**, 2222–2231 (2012).

[18] W. R. Gray Roncal, Z. H. Koterba, D. Mhembere, D. M. Kleissas, J. T. Vogelstein, R. Burns, A. R. Bowles, D. K. Donavos, S. Ryman, R. E. Jung, L. Wu, V. Calhoun, and R. J. Vogelstein, "MIGRAINE: MRI Graph Reliability Analysis and Inference for Connectomics," in *2013 IEEE Glob. Conf. Signal Inf. Process.* (IEEE, 2013) pp. 313–316.

[19] Lingzhong Fan, Hai Li, Junjie Zhuo, Yu Zhang, Jiaojian Wang, Liangfu Chen, Zhengyi Yang, Congying Chu, Sangma Xie, Angela R. Laird, Peter T. Fox, Simon B. Eickhoff, Chunshui Yu, and Tianzi Jiang, "The human brainnetome atlas: A new brain atlas based on connectional architecture," Cerebral Cortex **26**, 3508–3526 (2016).

[20] M. Bota and L. W. Swanson, "Online workbenches for neural network connections," J. Comp. Neurol. **500**, 807–814 (2007).

[21] S. N. Dorkenwald, P. J. Schubert, M. F. Killinger, G. Urban, S. Mikula, F. Svara, and J. Kornfeld, "Automated synaptic connectivity inference for volume electron microscopy," Nat. Methods **14**, 435–442 (2017).
