# Supplementary material for: Navigable maps of structural brain networks across species
Source: PLoS Comput Biol. 2020 Feb 3;16(2):e1007584. doi: 10.1371/journal.pcbi.1007584 (PMC7018228; doi:10.1371/journal.pcbi.1007584)
Supplement: S2 Appendix — (PDF) [file pcbi.1007584.s004.pdf]

CRITICAL ASSESSMENT OF THE DIFFERENT OPTIONS TO EVALUATE THE EFFICIENCY OF GREEDY ROUTING

Two metrics to measure the efficiency of greedy routing were introduced in Refs. [1, 2], respectively coined *efficiency ratio* and *GR score*, which are in fact the same quantity. They are related to the success rate and to the average stretch used in the main text. Although summarizing the navigability results in one single scalar may be appealing, these measures can lead to an ambiguity that the minimal "burden" of keeping track of two relatively simple measures does not justify. To further explain this position and to compare the different measures, let us introduce the following quantities.

- N: number of nodes in the network.
- N_g: number of greedy paths.
- N_s: total number of shortest paths. Note that $N_s = N(N-1)$ if the network is connected (i.e., one single component) which is the case in every connectomes considered in our study. Note also that $N_g \leq N_s$.
- Λ_{ij}^{bin}: length of the **greedy path** from node i to node j measured in terms of the **number of links crossed**. It is set to ∞ if no such path exists.
- Λ_{ij}^{*bin}: length of the **shortest path** from node i to node j measured in terms of the **number of links crossed**. It is set to ∞ if no such path exists. Note that $\Lambda_{ij}^{*bin} = \Lambda_{ji}^{*bin}$ if the network is undirected.
- Λ_{ij}^{geo}: length of the **greedy path** from node i to node j measured in terms of the **Euclidean/Hyperbolic distance** between each consecutive nodes on the path. It is set to ∞ if no such path exists.
- Λ_{ij}^{*geo}: length of the **shortest path** from node i to node j measured in terms of the **Euclidean/Hyperbolic distance** between each consecutive nodes on the path. It is set to ∞ if no such path exists. Note that $\Lambda_{ij}^{*bin} = \Lambda_{ji}^{*bin}$ if the network is undirected.
- $\Lambda_{ij}^{\dagger geo}$: length of the path whose length is the shortest, regardless of the number of nodes visited. Note that $\Lambda_{ij}^{\dagger geo} = \Lambda_{ji}^{\dagger geo}$ if the network is undirected.

In the main text, the success rate of greedy routing is defined as

$$R = \frac{N_g}{N_s} \tag{1}$$

and the average topological and geometrical stretches are defined as

$$S^{bin} = \frac{1}{N_g} \sum_{i,j\neq i}{}' \frac{\Lambda_{ij}^{bin}}{\Lambda_{ij}^{*bin}} = \frac{1}{N_g} \sum_{i,j\neq i}{}' S_{ij}^{bin} \ , \tag{2}$$

$$S^{geo} = \frac{1}{N_g} \sum_{i,j\neq i}{}' \frac{\Lambda_{ij}^{geo}}{\Lambda_{ij}^{*geo}} = \frac{1}{N_g} \sum_{i,j\neq i}{}' S_{ij}^{geo} \ . \tag{3}$$

where $S_{ij}^{bin/geo}$ denotes the stretch of an individual greedy path, and where the primed sum is over every ordered pairs i, j for which $\Lambda_{ij}^{bin} < \infty$, i.e. over all existing greedy paths.

The binary efficiency ratio introduced in Ref. [2] and the GR-score introduced in Ref. [1] are the same quantity (see also Refs. [3, 4]) and are defined as

$$E_R^{bin} = \frac{1}{N(N-1)} \sum_{i,j\neq i} \frac{\Lambda_{ij}^{*bin}}{\Lambda_{ij}^{bin}} = \frac{1}{N_s} \sum_{i,j\neq i} \frac{\Lambda_{ij}^{*bin}}{\Lambda_{ij}^{bin}} \ . \tag{4}$$

Note that the sum covers all possible paths and therefore considers greedy paths that do not exist. However, the length of these nonexistent greedy paths is set to $\Lambda_{ij}^{bin} = \infty$ and therefore contribute 0 to the sum. Assuming that the networks are connected—otherwise the theoretical maximum of E_R^{bin} would be less than 1—, we can rewrite the binary efficiency ratio as

$$E_R^{bin} = R \frac{1}{N_g} \sum_{i,j\neq i}{}' \frac{1}{S_{ij}^{bin}} \ . \tag{5}$$

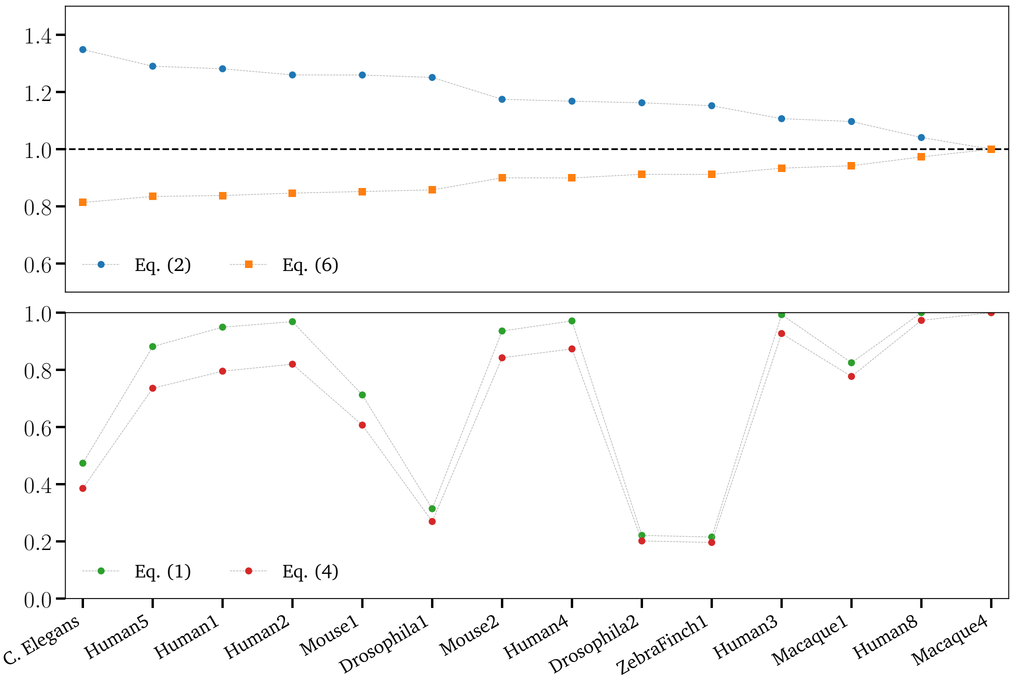

FIG. 1. Comparison of the value of various quantities defined in this Appendix.

The binary efficiency ratio can therefore be decoupled into two contributions: the success rate, R, and the average value of the reciprocal of the individual stretches

$$\frac{1}{N_g}\sum_{i,j\neq i}{}' \frac{1}{S_{ij}^{bin}} \ . \tag{6}$$

Because the lengths of greedy and shortest paths are typically of the same order of magnitude, Eqs. (2) and (6) mirror each other, and approach 1 respectively from above and from below as the greedy paths increasingly uncover the shortest paths of the network (see the top panel of Fig. 1). Both average values can therefore be seen as the two faces of a same coin, meaning that neither of them is more insightful than the other.

As shown on the bottom panel of Fig. 1, the behavior of the binary efficiency ratio is mainly governed by the value of the success rate, whose value is modulated by Eq. (6). Although appealing, the interpretation of this single-value summary of the performance of GR can be ambiguous. Consider for instance two networks, 1 and 2, for which

$$R^{(1)} = 0.95 \ ; \qquad \frac{1}{N_g^{(1)}}\sum_{i,j\neq i}{}'^{(2)} \frac{1}{S_{ij}^{bin,(1)}} = 0.684 \ ;$$

$$R^{(2)} = 0.70 \ ; \qquad \frac{1}{N_g^{(2)}}\sum_{i,j\neq i}{}'^{(1)} \frac{1}{S_{ij}^{bin,(2)}} = 0.929 \ .$$

Although both networks have roughly the same binary efficiency ratios, $E_R^{bin,(1)} \simeq E_R^{bin,(2)} \simeq 0.65$, their navigability are very different. In network 1, a greedy path is found for 95% of node pairs, but this high success rate comes at the expense that these paths are considerably longer than their respective theoretical minimum. In network 2, although GR was successful in only 70% of the time, it was able to uncover paths that were very close to the shortest paths of the network. This simple example illustrates how the measures proposed by Refs. [1, 2] can lead to ambiguous conclusions by coarse-graining the results one step too many, and therefore why we decided to present the success rates and the average stretches side by side. Moreover, considering the individual stretches allows us to look at their distribution (and transformation thereof), and therefore to conclude that the stretch of greedy paths in hyperbolic space are less dispersed around their average value than in Euclidean space.

A similar discussion can also apply to the distance-based efficiency ratio defined in Ref. [2] as

$$E_R^{dis} = \frac{1}{N(N-1)}\sum_{i,j\neq i}\frac{\Lambda_{ij}^{\dagger geo}}{\Lambda_{ij}^{geo}} = R\frac{1}{N_g}\sum_{i,j\neq i}\frac{1}{\Lambda_{ij}^{geo}/\Lambda_{ij}^{\dagger geo}} \ . \tag{7}$$

However, the principal difference with S^{geo} (geometrical stretch) is the quantity to which the length of greedy paths, Λ_{ij}^{geo}, are compared. In the case of S^{geo}, they are compared with the *length of the shortest paths*, Λ_{ij}^{*geo}. Doing so assesses whether GR, by taking into account distances, is able to find a path whose geometrical length (i.e., measured in terms of distances) is shorter than the shortest path found based on topological information only. In the case of E_R^{dis}, Λ_{ij}^{geo} is compared to the *shortest route in terms of distances only*, $\Lambda_{ij}^{\dagger geo}$, regardless of the number of nodes visited by the path. The distance-based efficiency, E_R^{dis}, therefore measures how close the greedy paths are to their respective theoretical minimum.

Both approaches consist in valid ways to characterize greedy paths. That being said, given that E_R^{dis} only confirms the conclusions drawn using E_R^{bin} in Ref. [2] (in the sense that it does not lead to new unexpected conclusions), it is therefore not expected that adding a third measure of the stretch in which Λ_{ij}^{geo} is compared to $\Lambda_{ij}^{\dagger geo}$ would bring critical new information.

---

[1] A. Cacciola, A. Muscoloni, V. Narula, A. Calamuneri, S. Nigro, Emeran A. Mayer, J. S. Labus, G. Anastasi, A. Quattrone, A. Quartarone, D. Milardi, and C. V. Cannistraci, "Coalescent embedding in the hyperbolic space unsupervisedly discloses the hidden geometry of the brain," arXiv:1705.04192 (2017), arXiv:1705.04192.

[2] C. Seguin, M. P. van den Heuvel, and A. Zalesky, "Navigation of brain networks." Proc. Natl. Acad. Sci. U. S. A. **115**, 6297–6302 (2018).

[3] A. Muscoloni and C. V. Cannistraci, "Navigability evaluation of complex networks by greedy routing efficiency," Proc. Natl. Acad. Sci. U. S. A. **116**, 1468–1469 (2019).

[4] C. Seguin, M. P. van den Heuvel, and A. Zalesky, "Reply to Muscoloni and Cannistraci: Navigation performance measures," Proc. Natl. Acad. Sci. U. S. A. **116**, 1470–1470 (2019).
