# Supplementary material for: Navigable maps of structural brain networks across species
Source: PLoS Comput Biol. 2020 Feb 3;16(2):e1007584. doi: 10.1371/journal.pcbi.1007584 (PMC7018228; doi:10.1371/journal.pcbi.1007584)
Supplement: S3 Appendix — (PDF) [file pcbi.1007584.s005.pdf]

CRITICAL GAP METHOD

The critical gap method (CGM) is a general technique that identifies non-overlapping communities in geometrically embedded networks based on the distances between nodes [1, 2]. In the case of hyperbolic embeddings, it first assigns each node to its own community, and then aggregates pairs of angularly consecutive nodes in a same community if their separation is smaller than a critical angular distance, $\Delta\theta_{\mathrm{c}}$. Note that the critical gap only defines the *boundaries* of the communities, meaning that only angularly consecutive nodes need to be separated by less than $\Delta\theta_{\mathrm{c}}$ to be part of a same community. In other words, three angularly consecutive nodes A, B and C, for which $\theta_{\mathrm{A}} < \theta_{\mathrm{B}} < \theta_{\mathrm{C}}$, will be in the same community if $\Delta\theta_{AB} < \Delta\theta_{\mathrm{c}}$ and $\Delta\theta_{BC} < \Delta\theta_{\mathrm{c}}$, independently of the value of $\Delta\theta_{AC}$ which can be larger than $\Delta\theta_{\mathrm{c}}$. Note that increasing $\Delta\theta_{\mathrm{c}}$ from 0 to 2π generates a set of N unique partitions of non-overlapping communities, where N is the number of nodes, and that we selected the partitions with the highest modularity [3].

[1] M. Á. Serrano, M. Boguñá, and F. Sagués, "Uncovering the hidden geometry behind metabolic networks," Mol. Biosyst. **8**, 843–850 (2012).

[2] G. García-Pérez, M. Boguñá, A. Allard, and M. Á. Serrano, "The hidden hyperbolic geometry of international trade: World Trade Atlas 1870-2013," Sci. Rep. **6**, 33441 (2016).

[3] M. E. J. Newman and M. Girvan, "Finding and evaluating community structure in networks," Phys. Rev. E **69**, 026113 (2004).
